# Epstein-Barr Virus LMP2A Reduces Hyperactivation Induced by LMP1 to Restore Normal B Cell Phenotype in Transgenic Mice

**DOI:** 10.1371/journal.ppat.1002662

**Published:** 2012-04-19

**Authors:** Alexandra C. Vrazo, Maria Chauchard, Nancy Raab-Traub, Richard Longnecker

**Affiliations:** 1 Department of Microbiology and Immunology, Feinberg School of Medicine, Northwestern University, Chicago, Illinois, United States of America; 2 Center for Global Health, Feinberg School of Medicine, Northwestern University, Chicago, Illinois, United States of America; 3 Lineberger Comprehensive Cancer Center, University of North Carolina at Chapel Hill, Chapel Hill, North Carolina, United States of America; University of Wisconsin-Madison School of Medicine and Public Health, United States of America

## Abstract

Epstein-Barr virus (EBV) latently infects most of the human population and is strongly associated with lymphoproliferative disorders. EBV encodes several latency proteins affecting B cell proliferation and survival, including latent membrane protein 2A (LMP2A) and the EBV oncoprotein LMP1. LMP1 and LMP2A signaling mimics CD40 and BCR signaling, respectively, and has been proposed to alter B cell functions including the ability of latently-infected B cells to access and transit the germinal center. In addition, several studies suggested a role for LMP2A modulation of LMP1 signaling in cell lines by alteration of TRAFs, signaling molecules used by LMP1. In this study, we investigated whether LMP1 and LMP2A co-expression in a transgenic mouse model alters B cell maturation and the response to antigen, and whether LMP2A modulates LMP1 function. Naïve LMP1/2A mice had similar lymphocyte populations and antibody production by flow cytometry and ELISA compared to controls. In the response to antigen, LMP2A expression in LMP1/2A animals rescued the impairment in germinal center generation promoted by LMP1. LMP1/2A animals produced high-affinity, class-switched antibody and plasma cells at levels similar to controls. *In vitro*, LMP1 upregulated activation markers and promoted B cell hyperproliferation, and co-expression of LMP2A restored a wild-type phenotype. By RT-PCR and immunoblot, LMP1 B cells demonstrated TRAF2 levels four-fold higher than non-transgenic controls, and co-expression of LMP2A restored TRAF2 levels to wild-type levels. No difference in TRAF3 levels was detected. While modulation of other TRAF family members remains to be assessed, normalization of the LMP1-induced B cell phenotype through LMP2A modulation of TRAF2 may be a pathway by which LMP2A controls B cell function. These findings identify an advance in the understanding of how Epstein-Barr virus can access the germinal center *in vivo*, a site critical for both the genesis of immunological memory and of virus-associated tumors.

## Author Summary

As a ubiquitous human pathogen, Epstein-Barr virus (EBV) infection is associated with several human B cell diseases characterized by inappropriate B cell activation and function, including infectious mononucleosis and certain cancers. EBV latent membrane protein 1 (LMP1) and 2A (LMP2A) hijack cell signaling pathways to alter B cell activation and function, and are detected in EBV-associated diseases. Defining the effect on B cell function when LMP1 and LMP2A are expressed together in the same cell is critical to understanding how EBV subverts normal B cell behavior before disease develops. Using transgenic mice, we have demonstrated that LMP2A dampens cellular proliferation and activation induced by LMP1, which may be due to the LMP2A-associated decrease in the levels of TRAF2, a signaling protein used by LMP1. LMP2A also allows B cells carrying LMP1 to enter the germinal center during an immune response, a site that gives rise to EBV-associated tumors in humans. In sum, this study highlights the biological outcomes of LMP1 and LMP2A co-expression in B cells and contributes to the knowledge of how EBV subverts normal B cell behavior before disease develops.

## Introduction

Epstein-Barr virus (EBV) is a B-lymphotropic gammaherpesvirus that establishes latent infection in over 90% of the world's population [1], [2]. Initial infection is usually asymptomatic if the virus is acquired during childhood. Following infection, EBV may persist for the life of the host in resting memory B cells where a limited number of viral genes are expressed [3]. EBV is also associated with a number of B cell malignancies, including Hodgkin's lymphoma, Burkitt's lymphoma, and post-transplant lymphoproliferative disorder, as well as epithelial malignancies such as nasopharyngeal carcinoma. *In vitro*, EBV has the unique ability of transforming primary human B cells into lymphoblastoid cell lines (LCLs) expressing the latency III program of gene products [4], including six EBV nuclear antigens (EBNA1, -2, -3A, -3B, -3C and -LP) as well as three latent membrane proteins (LMP1, -2A, and -2B), and multiple non-coding RNAs (EBERs and miRNAs).

LMP1 is a transmembrane protein with a cytoplasmic C-terminal tail that is transforming *in vitro* [5] and tumorigenic *in vivo* [6]. LMP1 signaling resembles that of the tumor necrosis factor superfamily member CD40, expressed on B cells; however, LMP1 signaling is constitutive and amplified [7]. LMP1 and CD40 signaling activate the B cell through downstream kinases and NF-κB, resulting in upregulation of surface costimulatory and adhesion molecules [8]–[10]. LMP1 signaling also plays a role in B cell survival by upregulating Bcl-2, A20 and Mcl-1 in human B cell lines and murine transgenic lymphomas [6], [11]–[13]. When expressed under the control of the immunoglobulin heavy chain (*IgH*) promoter and enhancer, LMP1 lineage 3 mice have normal lymphocyte populations, yet LMP1 predisposes to lymphoma development when aged [6], [14], [15]. Several transgenic models have demonstrated the immunomodulatory capacity of LMP1. LMP1 has been shown to block germinal center formation [10], [16], and to synergize with CD40 signaling to enhance proliferation and immunoglobulin production [16].

CD40 and LMP1 both utilize the tumor necrosis factor receptor-associated factor (TRAF) adaptor proteins for signaling [17], [18], but often in opposing ways in different experimental systems [7], [19], [20]. In knockout mouse embryonic fibroblasts, TRAF6, but not TRAF2 and TRAF5, was required for LMP1 signaling [21]. Compared to CD40, TRAF3 was shown to be preferentially used by LMP1 in murine B cells, and LMP1 could still signal in TRAF2 null cells while CD40 could not [7]. Conversely, a study using LCLs found that TRAF3 negatively modulated LMP1 activation of NF-κB [8]. Another study showed that TRAF2 was required by LMP1 in LCLs, and TRAF2 expression was controlled by LMP2A [22]. TRAF2 is critical for germinal center functions such as B cell proliferation, class switch recombination, and immunoglobulin secretion [23], yet findings from TRAF3 null mice suggest that TRAF3 prevents or delays germinal center entry [24]. Based on these arguments, we would hypothesize that LMP1 alters TRAF2 and TRAF3 regulation *in vivo* and that LMP2A might also affect TRAF regulation to indirectly modulate LMP1.

LMP2A is also capable of eliciting profound effects on B cell function *in vivo*. LMP2A is a functional homologue of the B cell receptor, and constitutively associates with Src family kinases through its N-terminal cytoplasmic tail to activate Ras/PI3K/Akt [25] and mTOR [26] to signal to NF-κB [22], [27]. LMP2A is not essential for LCL generation [28] nor B cell proliferation [29], but appears to promotes survival through upregulation of Bcl-X_L_ and survivin [30], [31]. Expression of LMP2A under the control of the immunoglobulin heavy chain (*IgH*) promoter and enhancer in the Tg6 lineage produces phenotypically normal B cells which express a BCR, and does not predispose to tumor development [31], [32]. In certain models, LMP2A overcomes the requirement for BCR expression [31]–[33], enhances antibody production and plasma cell frequencies [34], and alters tolerogenic signals induced through the BCR on the transgenic BCR^HEL^ background [27].

The patterns of LMP1 and LMP2A expression in humans can vary depending on the type of cell, tissue, or pathology analyzed. For example, EBNA1, EBNA2, LMP2A and the EBERs can be found in germinal center B cells, with varying detection of LMP1 [35], [36]. In some cases, LMP1 expression is restricted to B cells outside of germinal centers in the extrafollicular space [37], similar to observations in LMP1 transgenic mice [10], [16]. The bulk of EBV genomes in peripheral B cells are detected in class-switched memory B cells where latent gene expression is limited to EBNA1 [35], [38]–[40]. These findings have suggested a model whereby LMP1 and LMP2A promote terminal differentiation to a memory B cell in which EBV genomes can quiescently persist [3]. Therefore, we hypothesize that LMP2A could modify LMP1 signaling to allow the B cell to enter germinal centers.

Our hypothesis is supported by studies of LMP1 and LMP2A co-expression that indicated a role for LMP2A in altering LMP1 signaling through the TRAFs. In an early study, LMP2A expression reduced signaling through several receptors in EBV-negative Burkitt's lymphoma (BL) B cell line transfected with LMP1 and LMP2A [41]. Also, LMP2A decreased signaling from LMP1 by modulating the levels of TRAF2 and TRAF3 in BL cell lines [22]. However, these findings differ from studies with epithelial cell lines, where LMP2A appeared to stabilize LMP1, enhancing NF-κB activation [42]. These differential results in transformed epithelial and B cell lines, and the difficulties with studying latently EBV-infected B cells in humans warrants the study the effects of co-expression of LMP1 and LMP2A on TRAF levels *in vivo* using transgenic models.

To address whether LMP1 and LMP2A co-expression alters B cell maturation and function and to identify a role for LMP2A in modulation of LMP1, we generated double LMP1/2A B cell transgenic mice. Instead of LMP1 and LMP2A signals synergizing to enhance B cell proliferation, activation, and immunoglobulin secretion, we have identified that LMP2A modulates the LMP1-induced phenotype of the B cell following stimulation. The decrease in TRAF2, but not TRAF3, levels detected upon co-expression of LMP1 and LMP2A recapitulates *in vitro* findings with B cells lines in an animal model. Our results suggest a role for LMP2A in modulating the effect of LMP1 on B cell function *in vivo*, and have larger implications for the ability of Epstein-Barr virus in the subversion of normal B cell behavior before disease develops.

## Results

### LMP1 and LMP2A are expressed in B cells of transgenic mice

**Figure 1:**
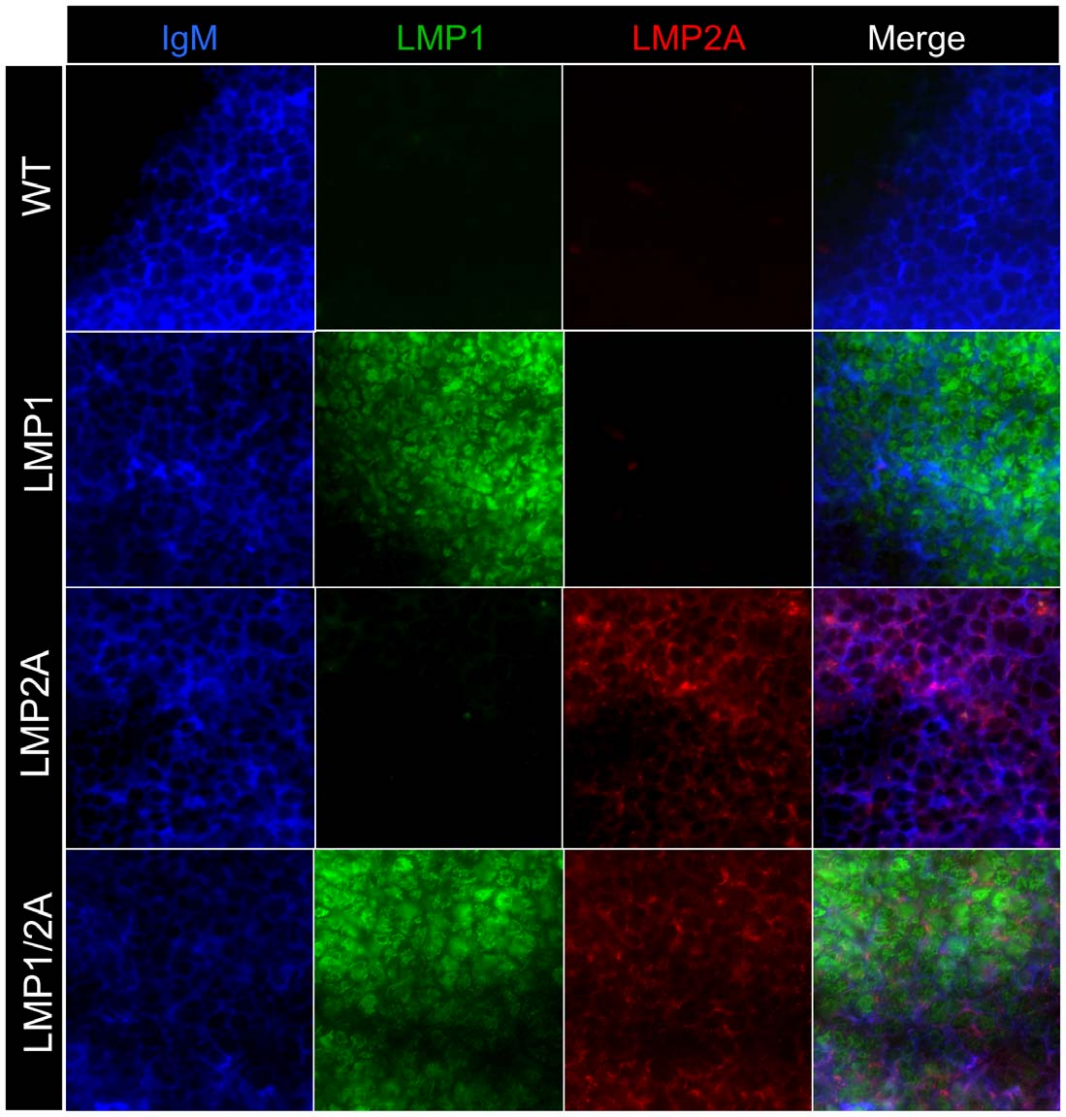
LMP1 and LMP2A are expressed in transgenic spleen. Immunofluorescence of LMP1, LMP2A and IgM-stained acetone-fixed spleen cryosections from one representative experiment. IgM staining is specific as shown by follicle border in WT panel; LMP1 and LMP2A staining is specific as shown by controls. Color code of antibodies used is shown. Magnification, 63×. *n*>3 mice per genotype.

To investigate the significance of expression of LMP1 and LMP2A in B cells, we crossed LMP1 and LMP2A single transgenic mice. Both lines express the transgene under the control of the *IgH* promoter and enhancer region, rendering transgene expression B cell-specific. The well-described LMP2A Tg6 line has no gross defect in B cell numbers, B cell development, or BCR expression [31], [32], [43]. In LMP1 lineage 3 mice, modest alterations have been described in B cell maturation in the periphery, as well as the ablation of germinal center (GC) formation in response to antigen [16]. We crossed LMP2A and LMP1 heterozygotes to obtain LMP1/2A transgenic mice, and used these mice and the LMP1, LMP2A or non-transgenic littermate controls (wild-type, WT) in each subsequent experiment. We first examined the expression of LMP1 and LMP2A protein in splenic B cells from the relevant genotypes as well as WT mice. Splenic cryosections from 8 week old mice were stained with antibodies to LMP1 and LMP2A and the B cell marker IgM. IgM staining was specific, as shown by the follicle border in the WT IgM panel (Top Left, Figure 1). IgM-positive B cells were also positive for LMP1 and/or LMP2A, and staining was specific, as shown by the lack of LMP1 or LMP2A staining in WT spleen (Figure 1). In all transgenic spleens, LMP1 or LMP2A-positive cells were located in IgM-positive B cell follicles at low power magnification (data not shown). These data confirm that LMP1 and LMP2A protein were expressed in B cells of LMP1/2A transgenic mice.

### Lymphoid organs of LMP1/2A animals are morphologically normal

**Figure 2:**
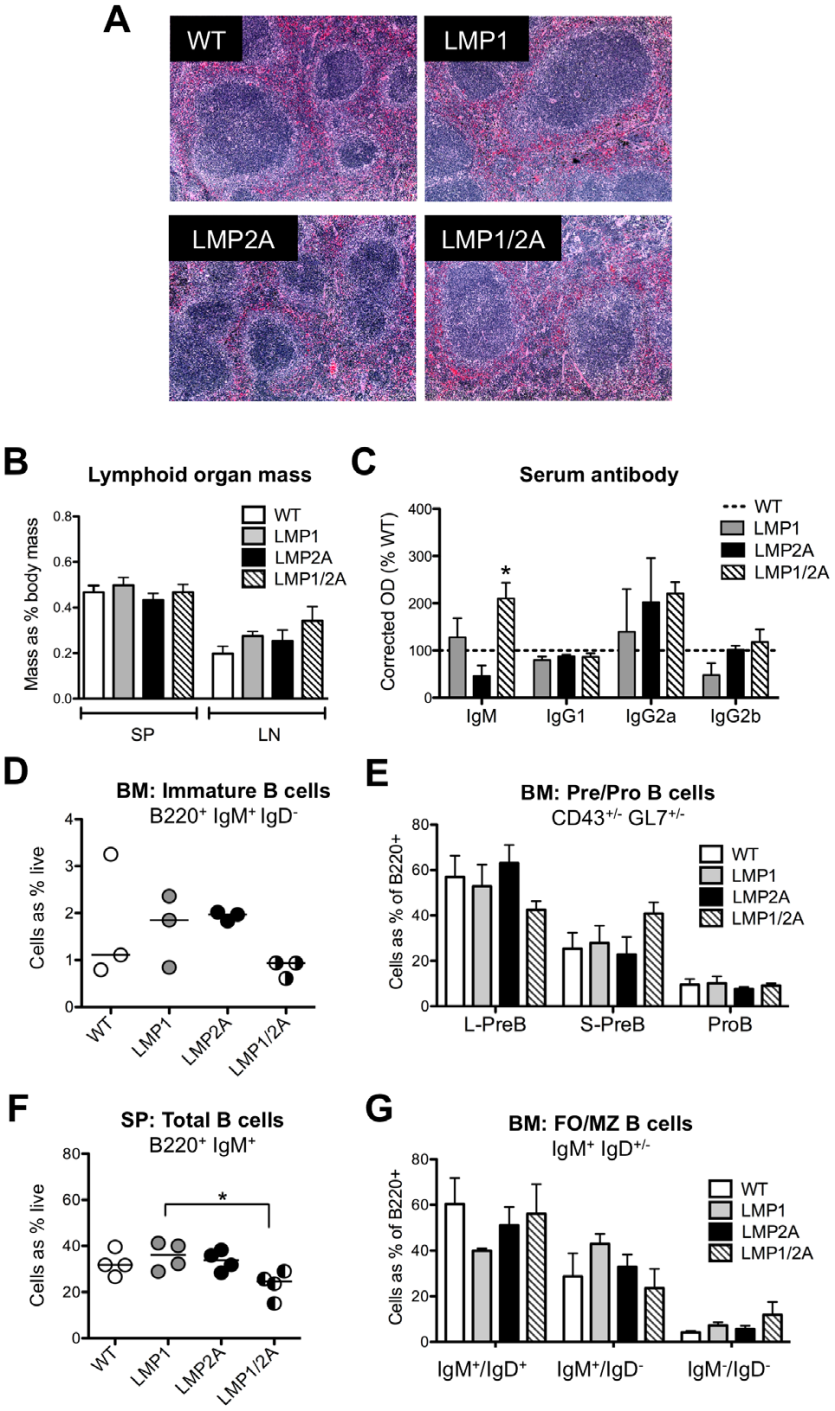
Spleen morphology, B cell maturation and antibody levels in LMP1/2A animals is similar to wildtype. (A) Spleen sections from 8 week old animals were isolated and fixed in 4% formalin. Paraffin-embedded sections were stained with H&E for identification of follicles and germinal centers. (B) Spleen, axillary and brachial lymph nodes were removed and weighed. (C) Sera of WT, LMP1, LMP2A and LMP1/2A mice was collected at 8 weeks of age and analyzed by ELISA for naïve Ig isotypes. Corrected OD of WT mice was set as 100%. (D–G) Single cell suspensions from lymphoid organs of WT, LMP1, LMP2A or LMP1/2A mice (6–8 weeks old) were surface stained with indicated antibodies and analyzed by flow cytometry. Frequencies of the live cell population (LiveDead stain negative) were averaged across 4 independent experiments. Either a representative experiment (D, F) or average of all mice used (E, G) is shown. (D) Immature bone marrow B cells (B220^+^/IgM^+^/IgD^−^). (E) Bone marrow large pre-B (L-PreB, CD43^+^/GL7^int^), small pre-B (CD43^+^/GL7^−^) and pro-B cells (CD43^−^/GL7^−^). (F) Total splenic B cells (B220^+^/IgM^+^). (G) Splenic follicular (FO) B cells (IgM^+^/IgD^+^), and marginal zone (MZ) B cells (IgM^+^/IgD^−^). BM, bone marrow; SP, spleen; LN, axillary and brachial lymph nodes. Data are represented as mean ± standard error. *n*>4 mice per genotype for all experiments; *, *P*<0.05, Student's *t* test.

We examined whether co-expression of LMP1 and LMP2A in B cells resulted in perturbation of normal splenic architecture, which has previously been described in LMP1 transgenic animals [6], [44]. We isolated spleens and axillary and brachial lymph nodes of mice at 8 weeks of age, weighed these organs, and stained spleen sections with H&E. In all genotypes, the splenic red and white pulp were well organized and follicles were clearly present with no spontaneous germinal centers observed (Figure 2A). The mass of lymph nodes and spleens of LMP1/2A animals was similar to WT, LMP1 and LMP2A animals (Figure 2B). Thus, in peripheral lymphoid organs, LMP1/2A co-expression did not alter follicle formation nor elicit spontaneous germinal center formation.

### Bone marrow B cell development is not altered by LMP1/2A co-expression

Since LMP1 and LMP2A act as constitutive signaling mimics of normal B cell signaling and LMP2A Tg6 mice have previously been described as having normal bone marrow B cell development [31], [32], we next examined whether expression of LMP1 and LMP1/2A altered B cell development in bone marrow. Bone marrow was flushed from tibia and femurs of 4, 6, or 8 week old mice, stained with fluorescent antibodies against B cell maturation markers, and analyzed by flow cytometry. Data from 8 week old mice is shown in Figure 2, although similar B cell populations were detected at 4 and 6 weeks (data not shown). Similar frequencies of immature B cells expressing a BCR of the IgM isotype (B220^+^/IgM^−^) were observed in mice of all genotypes (Figure 2D). Expression of LMP1 and/or LMP2A did not alter B cell maturation from pro-B to large and small pre-B, as shown by B220, CD43 and GL7 expression (Figure 2E). In addition, the frequencies of recirculating, mature B220^+^/IgM^+^/IgD^+^ B cells detected in bone marrow were similar across genotypes, suggesting that LMP1/2A co-expression does not alter mature B cell recirculation (Supp. Figure S1A). Taken together, these data suggest that LMP1/2A co-expression does not alter B cell ontogeny.

### LMP1/2A animals have normal peripheral B cell maturation and production of natural immunoglobulin

We next examined whether expression of LMP1 and LMP2A alters maturation of B cells by examining B cell populations in peripheral lymphoid organs. Upon exiting the bone marrow, immature bone marrow B cells home to the spleen via the blood, differentiating into mature follicular B cells which recirculate, or marginal zone B cells, which remain in the spleen. Spleen and axillary and brachial lymph nodes from mice at 8 weeks of age were isolated and single cell suspensions were prepared. Cells were stained with fluorescent antibodies against B cell maturation markers and analyzed by flow cytometry. In spleen, significantly fewer total B220^+^/IgM^+^ B cells were detected in LMP1/2A spleen compared to LMP1, LMP2A and WT spleen (Figure 2F). Lower, but not statistically significant, frequencies of mature follicular (FO) B cells (B220^+^/IgM^+^/IgD^+^) were observed in LMP1 spleen (Figure 2F), and this was consistent with a slight increase in marginal zone (MZ) B cells (B220^+^/IgM^+^/IgD^−^) in LMP1 spleen, while LMP2A and LMP1/2A B cell populations appeared more similar to WT (Figure 2G). The frequency of splenic B1 B cells (Figure S1B) and T cells (Figure S1C) was not altered by expression of LMP1 and/or LMP2A when compared to WT. Next, we assessed frequencies of total (B220^+^/IgM^+^), FO B cells (B220^+^/IgM^+^/IgD^+^) and MZ B cells (B220^+^/IgM^+^/IgD^−^) in axillary and brachial lymph nodes. The expression of LMP1 and/or LMP2A did not alter the frequencies of total lymph node B cells (Figure S1D), MZ or FO B cells (Figure S1E), or CD4^+^ and CD8^+^ T cells (Figure S1F).

Because LMP1/2A animals did not demonstrate gross alteration of B cell maturation, we postulated that the levels of natural immunoglobulin in LMP1/2A animals would be similar to controls. We assessed the levels of immunoglobulin in serum from 8 week old naïve animals by isotype-specific ELISA. Levels of IgM in LMP1/2A serum were significantly increased (*p*<0.05) over LMP1, LMP2A and WT animals, whereas levels of IgG1, IgG2a and IgG2b were similar across all genotypes (Figure 2C).

### Germinal center formation is rescued in LMP1/2A animals following immunization with thymus-dependent antigen

**Figure 3:**
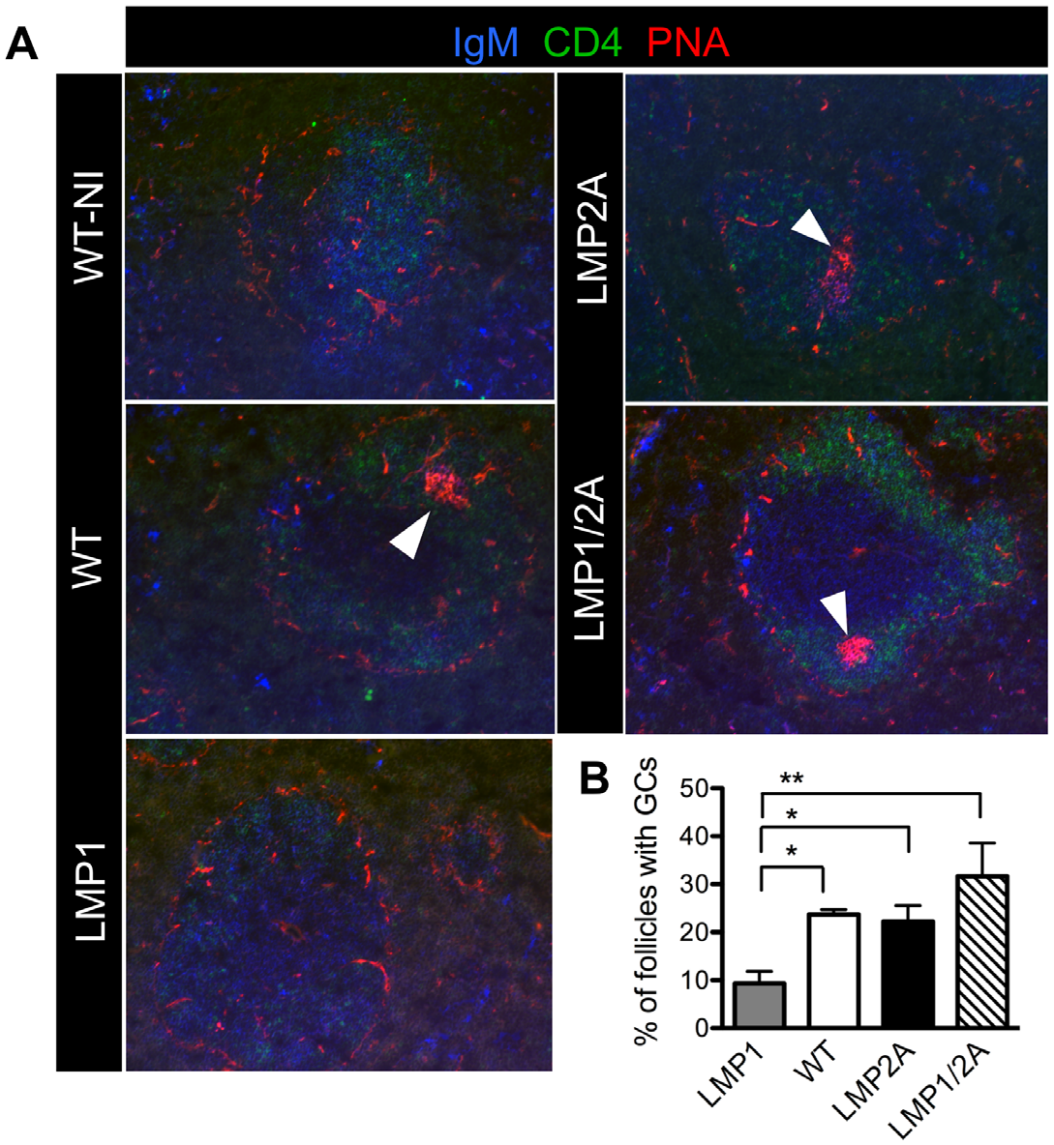
LMP2A co-expression rescues LMP1 impairment of germinal center formation. (A) Spleen cryosections from immunized WT, LMP1, LMP2A and LMP1/2A mice at Day 7 of the primary response and a WT non-immunized control (WT-NI) were stained with the indicated reagents. A representative image is shown from 3 experiments with at least 3 mice per genotype. Arrows indicate location of germinal center (PNA^+^/IgM^+^) in follicles. (B) Percentage of follicles containing germinal centers calculated from blinded counts of two serial sections for each mouse with 3–5 mice per genotype. Data are represented as mean ± standard error. **, *P*<0.01, Type I ANOVA and Dunnett's multiple comparison test.

To assess whether LMP2A alters the ability of LMP1-expressing LMP1/2A B cells to enter GC in response to antigen, we immunized mice with the hapten TNP_24_-KLH and isolated spleen at Day 7, as the GC response peaks between Days 7 and 10. Splenic cryosections were stained for the GC B cell markers PNA and IgM, as well as CD4 for T helper cells to demarcate follicles (Figure 3A). The location of GC in follicles was confirmed by staining separate sections for the germinal center marker GL7 (data not shown). PNA^+^/IgM^+^ GC were observed in spleens of WT mice, surrounded by a network of CD4+ T helper cells (Figure 3A). PNA-positive GC per follicle were counted and the percentage of follicles containing GC was enumerated (Figure 3B). As previously shown [16], GC were rarely detected in LMP1 spleen (Figure 3A), and the frequency of GC per follicle was significantly decreased in LMP1 spleen to less than half that of WT (*p*<0.05) (Figure 3B). Similar to WT, LMP2A-expressing B cells were able to enter GC. Intriguingly, LMP1/2A-expressing B cells were able to form GC at a similar frequency per follicle compared to LMP2A or WT animals, suggesting that the LMP2A signal restores normal GC development and allows LMP1-expressing B cells to enter GC.

### High affinity antigen-specific antibody is generated in LMP1/2A animals

**Figure 4:**
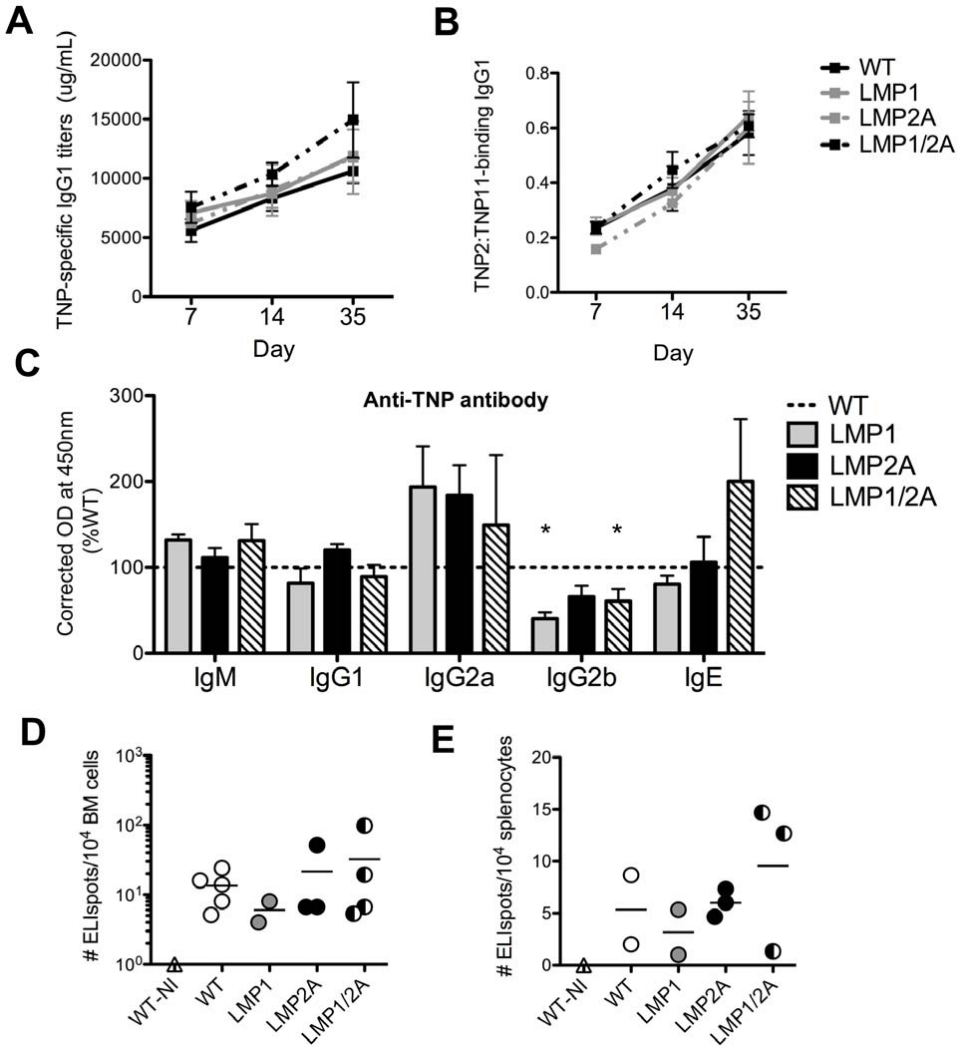
LMP1/2A co-expression maintains antibody generation and frequency of plasma cells similar to wildtype. (A–C) Sera of WT, LMP1, LMP2A and LMP1/2A mice was collected from animals immunized with 100 µg TNP_24_-KLH at Day 7, 14 and 35 of the primary response, and analyzed by ELISA for (A) TNP-specific IgG1 titers over time, and (B) for affinity (ratio TNP_2_∶TNP_11_ binding) over time, and (C) for TNP-specific isotype levels at Day 35. Data are represented as mean ± standard error of 3–5 mice per genotype over 3 independent experiments. (D–E) Comparison of the number of TNP-specific IgG1 ELIspots per 10^4^ cells at Day 7 of the secondary response from bone marrow (D) or spleen (E). A representative image is shown from 4 experiments with 3–5 mice per genotype. Horizontal line represents mean. *, *P*<0.05, Type I ANOVA and Dunnett's multiple comparison test.

Previously, it was shown that despite the inability of LMP1 B cells to participate in the GC reaction, LMP1 expression maintains the production of high-affinity class switched antibody at levels similar to WT animals [16]. We assessed the kinetics of TNP-specific IgG1 production in serum of immunized LMP1/2A mice compared to single LMP1, LMP2A, and WT animals during the primary response. Between Day 7 and Day 35 following immunization, the levels of TNP-specific IgG1 increased with similar kinetics among all genotypes and was maximal by Day 35 (Figure 4A). The germinal center response is also critical for affinity maturation. TNP-specific antibodies were tested by ELISA for high or low affinity for TNP by binding to low-density or high-density hapten, respectively. Serum isolated at Days 7, 21 and 35 following immunization was assayed for high and low affinity IgG1. The ratio of TNP_2_∶TNP_11_-binding IgG1 increased with similar kinetics over time for each genotype, and was not altered by expression of LMP1 or LMP2A either alone or in combination (Figure 4B).

We also explored the possibility that LMP1 and LMP2A co-expression in B cells responding to antigen might enhance signals for class switching to IgG and IgE, as has been shown in LMP1-expressing cell lines [45]. By ELISA, we compared serum levels of TNP-specific immunoglobulin at Day 35 of the primary response. IgM, IgG1 and IgE levels were similar in LMP-expressing mice compared to WT. TNP-specific IgG2a levels in LMP1, LMP2A, and LMP1/2A mice were elevated compared to WT (Figure 4C). Conversely, the expression of LMP1 and LMP1/2A decreased TNP-specific IgG2b levels in serum; for LMP1 and LMP1/2A mice, this decrease was significant when compared to WT (*p*<0.05). The physiological significance of the decrease in IgG2b in LMP1 and LMP1/2A animals is unclear at present.

### LMP1/2A animals generate normal frequencies of plasma cells during the secondary response

It has been proposed that co-expression of LMP1 and LMP2A during the GC reaction may drive antigen-specific B cells to become memory B cells by augmenting BCR and CD40 signals [3]. If higher percentages of memory B cells were present in LMP1/2A transgenic mice, an increased frequency of antigen-specific plasma cells may be detected following secondary immunization. Thus, the ability of LMP1, LMP2A, and LMP1/2A to alter plasma cell generation during the secondary immune response was investigated using TNP_24_-KLH. Mice were boosted with TNP_24_-KLH at Day 50 following primary immunization, and TNP-specific IgG1 antibody-secreting cells (ASCs) were enumerated in bone marrow and spleen by ELIspot on Day 7 after boost. A representative experiment is shown (Figure 4D–E). As expected, levels of bone marrow plasma cells were tenfold higher in bone marrow than spleen among all genotypes, reflecting the ability of plasma cells to home to bone marrow following generation in secondary lymphoid tissues (Figure 4D). The frequencies of TNP-specific IgG1^+^ ASCs in LMP1/2A mice were comparable to controls in both bone marrow (Figure 4D) and spleen (Figure 4E), suggesting that LMP1, LMP2A, and LMP1/2A do not alter the frequency of IgG1^+^ ASC nor the ability of plasma cells to home from spleen to bone marrow during the secondary response.

### LMP1/2A has heterogeneous effects on B cell activation following BCR stimulation

**Figure 5:**
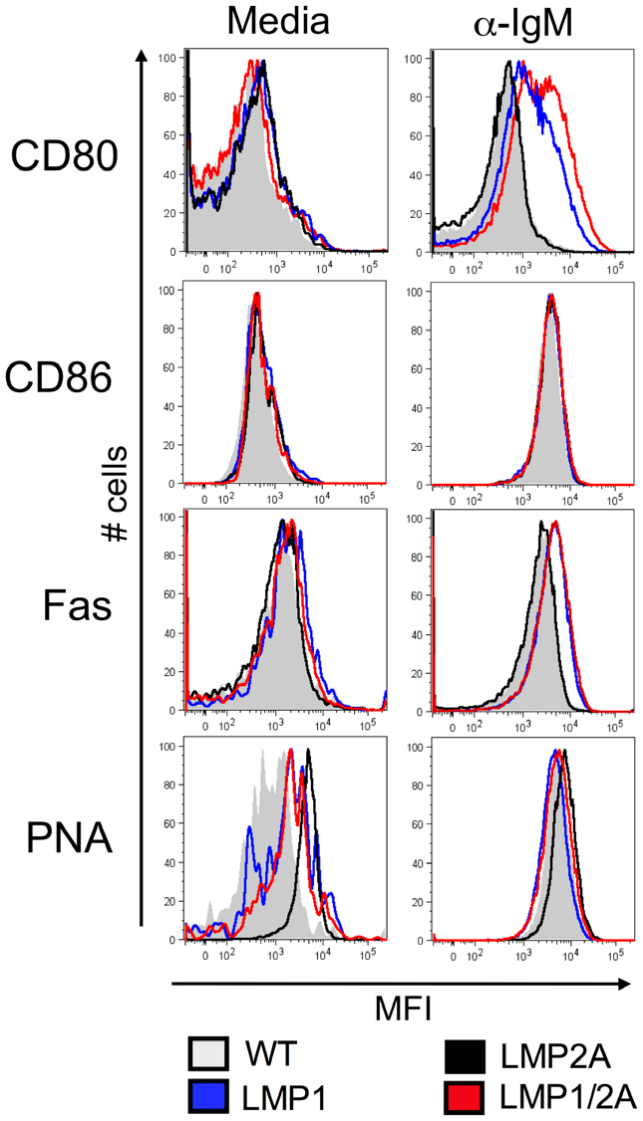
LMP1/2A has heterogeneous effects on B cell activation following BCR stimulation compared to controls. Splenic CD43^−^ B cells were purified by magnetic beads and incubated for 72 hours in complete media (Left panel) or with 10 µg/mL anti-IgM (F(ab′)_2_) (Right panel). Cells were stained for surface activation marker expression as indicated and analyzed by flow cytometry. One representative experiment of 4 experiments is shown with >4 mice per genotype.

Next, we tested whether co-expression of LMP2A altered the expression of B cell surface activation and co-stimulation markers *in vitro* on LMP1-expressing cells. Following antigen exposure, B cells upregulate the co-stimulatory molecules CD80 and CD86, which interact with T cell ligands to elicit secondary activation signals critical for GC formation [46], [47]. Once B cells enter the GC, they upregulate Fas and bind PNA at higher levels than non-GC B cells [48], [49]. Splenic B cells were purified by negative selection using CD43^+^ microbeads to generate a >95% pure population of resting naive B cells. B cells were stimulated with anti-IgM to crosslink B cell receptors for 72 hours, due to maximal upregulation of all markers by this time [50]–[52]. B cells were surface stained for expression of CD80, CD86, and Fas, and with PNA-FITC, and were analyzed by flow cytometry by gating on live B220^+^ cells. Resting B cells from all genotypes appeared similar in expression levels of all markers, although LMP1 B cells expressed slightly higher surface levels of Fas (Figure 5), confirming previous observations with this transgenic line [16]. Upon BCR cross-linking, all genotypes upregulated expression of all markers but to different levels. LMP1 and LMP1/2A further upregulated CD80 and Fas upon BCR crosslinking compared to WT and LMP2A alone. The intensity of PNA staining on LMP1 B cells was decreased compared to WT, LMP2A and LMP1/2A controls. In sum, LMP1 expression alone or with LMP2A appears to enhance cell surface expression of CD80 and CD86 following B cell stimulation, but decreases levels of PNA following IgM stimulation compared to WT and LMP2A alone.

### LMP2A dampens LMP1-driven hyperproliferation following B cell stimulation

**Figure 6:**
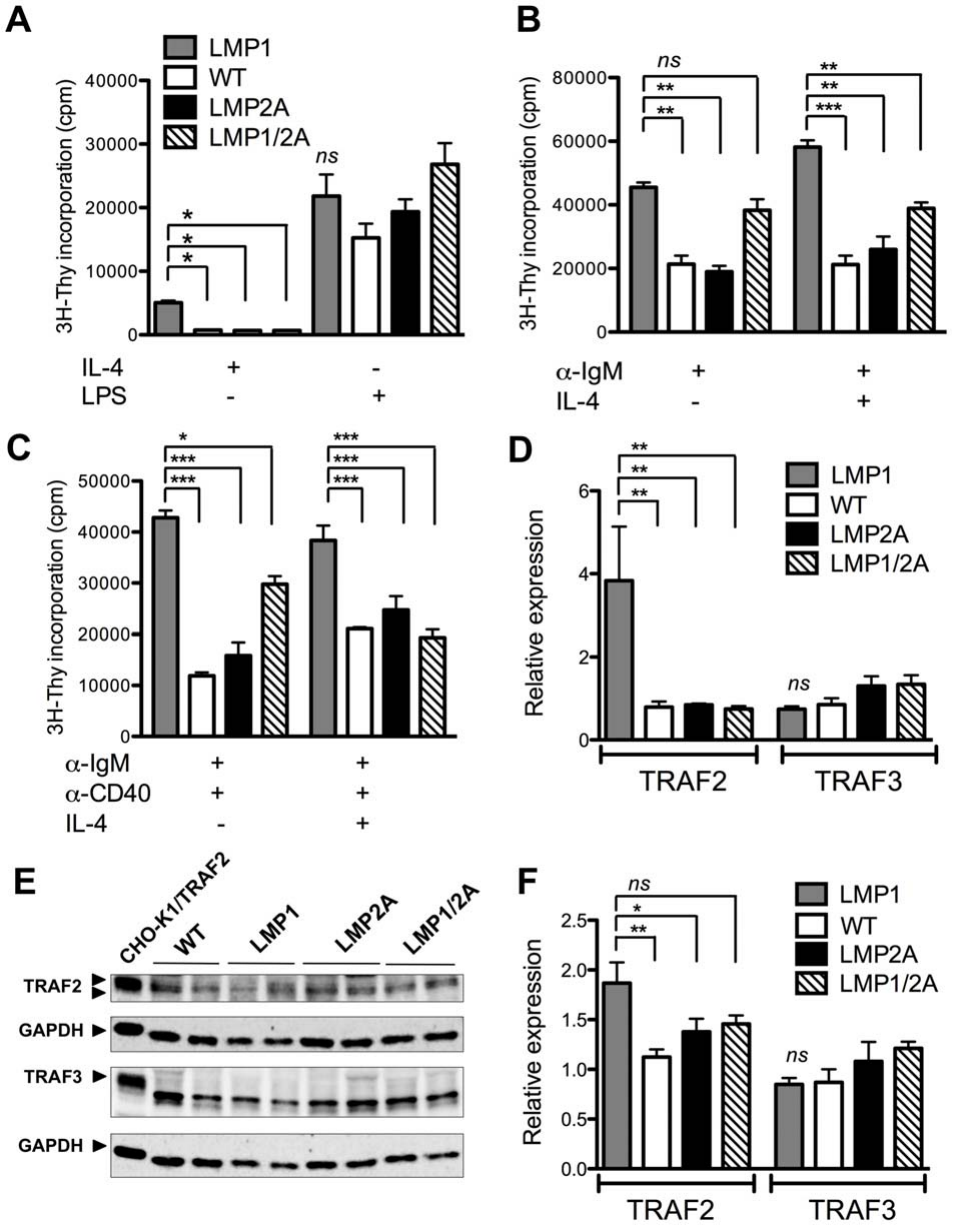
Co-expression of LMP1/2A decreases B cell proliferation and TRAF2 levels compared to LMP1. (A–C) Purified splenic CD43^−^ B cells were stimulated with combinations of IL-4, LPS, anti-IgM and anti-CD40. Proliferation as measured by ^3^[H]-thymidine incorporation is shown for 8 experiments with 8 mice per genotype. (D) Levels of TRAF2 and TRAF3 message were compared by RT-PCR using the ΔΔC_T_ method with HPRT as a housekeeping gene. Fold change in TRAF2 and TRAF3 expression in transgenic B cells compared to wildtype is shown for at least 4 experiments with 9 mice per genotype. (E) Immunoblot analysis shows TRAF2 (∼53 kDa), TRAF3 (∼62 kDa) and GAPDH (∼38 kDa) expression in purified B cells from WT, LMP1, LMP2A and LMP1/2A mice and TRAF2-expressing CHO-K1 cells. Each lane represents a single mouse, and the data are representative of several immunoblot analyses with between 6 and 12 mice per genotype. (F) Quantification of TRAF2 signal normalized to GAPDH and wildtype TRAF2 signal is shown as an average of all mice from each genotype. For a description of quantification, see Methods. LMP1 bars have been placed first as LMP1 B cells gave the largest responses. Data are represented as mean ± standard error. *, *P*<0.05; **, P<0.01; ***, *P*<0.001 by one way ANOVA.

Next, we wanted to assess whether LMP1/2A co-expression influenced *in vitro* B cell proliferation in response to BCR and T cell signals, as both LMP1 and LMP2A have been shown to influence proliferation in the presence of antigens, mitogens, or oncogene overexpression [16], [34], [53]–[55]. Purified resting CD43^−^ B cells were stimulated with optimized concentrations of anti-IgM, anti-CD40 and/or recombinant murine IL-4. LPS was used as a BCR and CD40-independent positive control. Proliferation of B cells was assessed by incorporation of ^3^[H]-thymidine at 72 hours of culture. Under all stimuli, LMP1 promoted B cell hyperproliferation and recapitulated the ability to proliferate with the T helper cell cytokine IL-4 that has been observed elsewhere [16], while LMP2A and LMP1/2A did not synergize with IL-4 (Figure 6A). B cells from all genotypes proliferated in response to LPS, but there was no significant difference in proliferation level (Figure 6A). In the presence of a BCR agonist, LMP1/2A B cells proliferated at the same level as LMP1 B cells (Figure 6B), indicating that the LMP2A signal did not synergize with nor impair BCR-induced proliferation, similar to previous results with LMP2A transgenic mice [25], [27]. However, when stimulated with a BCR agonist in the presence of IL-4, LMP1/2A B cells exhibited a lower level of proliferation compared to LMP1 alone (Figure 6B). We also observed the dampening effect of LMP2A and intensification of this effect by IL-4 when LMP1/2A B cells were stimulated with signals mimicked by LMP1 and LMP2A (i.e. the BCR and CD40) (Figure 6C). These data suggest that LMP2A co-expression decreased the hyperproliferation driven by LMP1, and that this normalizing phenotype in LMP1/2A B cells is more strongly promoted in the presence of IL-4 stimulation.

### LMP2A decreases the LMP1 signaling mediator TRAF2 in resting LMP1/2A B cells

The cytoplasmic C terminal activating domain-1 (CTAR1) of LMP1 directly recruits TRAF2 and TRAF3 to elicit proliferative effects on B cells, [8], [9], [17], [44] and LMP2A has been shown to decrease TRAF2 and TRAF3 expression in B cell lines expressing LMP1 [22]. To examine mRNA levels of TRAF2 and TRAF3 in resting CD43^−^ splenic B cells, we prepared cDNA from isolated CD43^−^ splenic B cells and amplified TRAF2 and TRAF3 message by RT-PCR. In resting LMP1 B cells, TRAF2 was upregulated to approximately four-fold compared to WT (Figure 6D). LMP2A expression decreased TRAF2 mRNA by half compared to WT, which was also observed in LMP1/2A-expressing B cells. The levels of TRAF3 were not significantly altered by either single or co-expression of LMP1 and LMP2A.

To assess whether the effects on TRAF2 and TRAF3 at the mRNA level were recapitulated at the protein level, we carried out TRAF2 and TRAF3 immunoblots on lysates prepared from purified B cells and CHO-K1/hTRAF2 cells using the housekeeping protein GAPDH as a loading control (Figure 6E). The low levels of expression of TRAF2 in purified B cells have been seen in other murine models [56], [57]. To confirm TRAF2 levels, we used an additional anti-TRAF2 antibody that gave similar results (Figure S2). Quantitation revealed that TRAF2 levels were increased up to twofold in LMP1 B cells (Figure 6F) while TRAF2 levels in LMP2A and LMP1/2A B cells were similar to WT. Expression of LMP1, LMP2A, and LMP1/2A did not appear to significantly alter TRAF3 protein levels. [56], [57]. In sum, LMP1 appears to increase mRNA and protein levels of TRAF2, but not TRAF3, and this increase is reversed when LMP2A is co-expressed with LMP1. Hence, in resting B cells, LMP2A expression appears to regulate levels of TRAF2, a molecule critical for LMP1 signaling.

## Discussion

Our results describe LMP2A as a regulator of LMP1-induced B cell hyperactivation during LMP1/2A co-expression in B cells in an animal model of EBV latency. Specifically, we found that in LMP1/2A animals: (1) LMP2A dampened LMP1-mediated hyperproliferation in response to mitogenic stimuli; (2) LMP2A expression allowed LMP1-expressing B cells to enter germinal centers consistent with decreased proliferation; (3) LMP2A decreased levels of TRAF2, required by LMP1 for signaling to NF-κB. The finding that B cell maturation is not perturbed in naïve LMP1/2A animals in the absence of a strong antigenic signal supports this conclusion.

Many studies of LMP1 and LMP2A expression in cell lines and transgenic mouse models of disease have indicated that these viral proteins can elicit profound effects on B cell function. Our study is the first to explore the effect of co-expressing both LMP1 and LMP2A in the same naïve B cell from early stages of development, and indicates that LMP1/2A-expressing B cells develop and function normally in naïve mice without driving spontaneous B cell proliferation in the absence of antigen. Once stimulated by antigen, LMP1/2A normalized responses in terms of germinal center physiology and antibody production, as well as production of normal plasma cell frequencies during the secondary response. Whether LMP1/2A can alter plasma cell numbers during the primary response remains to be determined, although antibody titers in LMP1/2A animals were similar to control animals during the primary response.

Cooperation of LMP1 and LMP2A signaling has been reported *in vitro* [42], however, we did not detect evidence of synergistic activity of LMP1 and LMP2A in our model, as demonstrated by no acceleration of mortality (data not shown), the dampening effect on B cell proliferation in LMP1/2A B cells, and the downmodulatory effect of LMP2A on TRAF2 levels. Our findings are the first description of LMP2A alteration of LMP1 signaling by TRAF2 modulation in an animal model, and support similar findings in EBV-positive nasopharyngeal carcinoma and Burkitt's lymphoma cell lines [22], [41], [58].

Previous studies of EBV-positive or LMP1-transfected tumor cell lines proposed that LMP1 perturbs TRAF regulation in order to enhance NF-κB activity [8], [9], [21]. One study identified that protein levels of TRAF2 were increased in transformed B cell lines when LMP1 was expressed, but that co-expression of LMP2A decreased TRAF2 levels to normal [22]. These data are consistent with our findings that LMP2A decreases TRAF2 transcript and protein levels in resting LMP1/2A B cells *in vivo*. The *in vivo* effect of the two-fold increase in TRAF2 detected in LMP1 mice is currently unclear. While TRAF2 transmits signals from TNF family receptors to AP-1 and NF-κB, studies from TRAF2 deficient mice have revealed potentially conflicting roles in activation of the canonical and noncanonical NF-κB pathways [56], [57], [59], [60]. Future studies of TRAF2 requirement for NF-κB activation and the effect on the B cell phenotype are warranted, especially with regards to the ability of TRAF2 to promote synergy between BCR and CD40 signals, and to degrade TRAF3 during CD40 signaling [61]. In addition, the effects on LMP1 localization and turnover when LMP2A is co-expressed necessitate additional investigation, as previous data suggested that LMP1 turnover could be altered by LMP2A co-expression in epithelial cell lines [42], which may alter TRAF2 recruitment by LMP1.

The most striking finding during the T cell-dependent immune response in LMP1/2A mice is the restoration of germinal center frequencies similar to LMP2A and WT mice, suggesting that LMP1 signals impeding GC formation may be overcome in the presence of LMP2A. Signals from activated CD40 through TRAF2 and NF-κB are critical for GC-associated functions, including B cell proliferation, class switch recombination, and immunoglobulin secretion [23]. Although the two-fold increase in TRAF2 message in LMP1 B cells is difficult to reconcile with the finding that LMP1 B cells are impaired in GC entry, a possible explanation may be that an overly strong CD40 signal prevents B cells from entering germinal centers. In support of this hypothesis, one study suggested that overly strong CD40 signaling downregulated the master germinal center regulator, BCL-6 [62], while another study found that overexpression of CD40 drives B cells to exit the germinal center to memory [63]. While BCL-6 levels and activity have not been assessed in this study, several lines of evidence support LMP1 repression of BCL-6 expression in EBV-positive Hodgkin's lymphoma cells [64], [65]. While LMP2A appears to have a more variable effect on BCL-6 in human B cell lines and primary transgenic murine B cells [30], [33], other evidence supports a role for LMP2A in promoting a germinal center-like cellular environment in LMP1/2A B cells. Microarray analysis of LMP2A transgenic B cells and LMP2A-positive LCLs indicated that LMP2A induces a gene expression pattern that resembles that detected in GC centroblasts [30]. Thus, it is possible that during the response to antigen, LMP1 and CD40 signaling together provide an overly strong CD40 signal, downregulating BCL-6 and diverting B cells from entering the germinal center, which may be rescued by the ability of LMP2A to induce a GC-like gene expression program. Evidence suggests that LMP1 and LMP2A also alter B cell cytokine profiles, which may have global effects on gene transcription, warranting future study of the alteration in cytokines and global gene expression induced during LMP1/2A-co-expression. Taken together, our findings suggest a model whereby LMP1/2A co-expression does not provide a synergistic signal for B cell activation, but instead normalizes B cell function by allowing B cells to enter the germinal center in a manner that may be advantageous to the virus.

As LMP1 and LMP2A confer survival and proliferative functions to latently EBV-infected B cells *in vitro*, several groups have suggested that these characteristics may allow latently infected human B cells to survive the germinal center reaction in order to enter the memory pool [3], [35], [39]. However, conflicting reports exist on the detection of LMP1 and LMP2A in human GC B cells [36], [37]. The entry of LMP1/2A-expressing B cells into germinal centers described herein is notable, as it is the first time that detection of LMP1/2A-expressing B cells in germinal centers of latently infected humans can be recapitulated in a mouse model. Similar to findings with latently-infected humans and LMP1 transgenic mice, we have confirmed that LMP1 B cells are defective in the ability to enter GC [16], [37], although the location of LMP1 B cells during an immune response and the nature of the signals received that generate high-affinity antibody, memory and plasma B cells are not well-defined. Our findings suggest that LMP2A expression in LMP1/2A B cells alters B cell biology by allowing LMP1-expressing B cells to transit the germinal center and successfully become memory cells, based on the finding that LMP1/2A B cells differentiate into plasma cells at the same frequency as wild-type animals during a secondary immune response.

As highly-proliferative LMP1-expressing B cells responding to antigen are more at risk of recognition by CD8^+^ T cells, the expression of LMP2A with LMP1 could be a viral strategy to normalize B cell physiology during the response to antigen. If this were the case, the latently-infected GC B cell could potentially access the memory compartment. The presence of LMP1/2A-expressing B cells in the germinal center has other implications for germinal center-derived neoplasms that express LMP1 and LMP2A, such as Hodgkin's lymphoma. It is plausible that a B cell that would normally apoptose due to a selection defect during GC transit might be rescued by LMP1/2A co-expression, allowing aberrant B cell activation, proliferation, and survival, which are hallmarks of EBV-associated germinal center-derived tumors. As such, our findings underscore the utility of the LMP1/2A model in providing novel insights as to the behavior of LMP1/2A-expressing B cells *in vivo* before the development of overt disease.

## Materials and Methods

### Ethics statement

This study was carried out in strict accordance with the recommendations in the Guide for the Care and Use of Laboratory Animals of the National Institutes of Health. The protocol was approved by and all experimental procedures were in compliance with the Institutional Animal Care and Use Committee of Northwestern University. Where indicated, procedures were performed under isoflurane anesthesia and all efforts were made to minimize suffering.

### Generation of LMP1/2A double transgenic mice and PCR genotyping

LMP1 lineage 3 heterozygotes (LMP1) [6] were backcrossed to C57BL/6 mice and crossed with LMP2A Tg6 heterozygotes [32] to generate double transgenic LMP1/2A mice. Both transgenes are driven by an *IgH* promoter and enhancer region. Expression of transgenes was confirmed by multiplex PCR on genomic DNA isolated from tail snips [27]. Primers used were OL106 (TACCCTGAGCTTCAGTTCTGCACC) and OL107 (TGACTGTGGGAACTGCTGAACTTT) (RAG control, 560 bp), LMP2A-RC-F2 (TCTTCTGTTTGCATTGCTGG) and LMP2A-RC-R2 (TCCAGAAAACATGTGGCAAA) (LMP2A, 404 bp), LMP1-AA-F1 (ATGGCCAGAATCATCGGTAG) and LMP1-AA-R1 (CACACCCCCTTTCCCTTACT) (LMP1, 490 bp). LMP1 and LMP2A expression in transgenic B cells was confirmed by immunofluorescence on spleen sections. All non-transgenic littermates are referred to as wildtype (WT) mice.

### Antibodies and reagents

Antibodies against LMP1 and LMP2A included rat 14B7 (LMP2A) and rabbit Lympa-1 (LMP1; gift of Dr. K. Izumi, UT Health Center, San Antonio). Monoclonal antibodies against mouse IgM, IgD, IgG1, IgG2b, IgG2a, B220, CD19, GL7, CD21, CD23, CD43, CD138, CD38, CD4, and CD8 were purchased from BD Biosciences (San Jose, CA). Antibodies used in immunoblotting included TRAF2 C-20 (Santa Cruz, Santa Cruz, CA), TRAF2 #4712, TRAF3 #4729 (Cell Signaling, Danvers, MA) and GAPDH ab8245 (Abcam, Cambridge, MA). Germinal center staining was carried out with PNA-biotin (Vector, Burlingame, CA). Viability reagents included Live/Dead Fixable Violet (Invitrogen, Carlsbad, CA). Secondary reagents included streptavidin-HRP (GE Healthcare, Piscataway, NJ), streptavidin-AP (Vector, Burlingame, CA), streptavidin-Alexa 488 (Invitrogen, Carlsbad, CA), goat anti-rat Cy3, anti-rabbit Cy5 (Jackson ImmunoResearch, West Grove, PA) and goat anti-mouse IRDye 800 and goat anti-rabbit IRDye 680 (LiCor, Lincoln, NE).

### Isolation of primary lymphoid cells

Single cell suspensions of bone marrow, spleen and lymph node cells were prepared as previously described [27], [32]. For flow cytometry, cells were washed in cold FACS buffer (1% fetal bovine serum [FBS] in 1× phosphate buffered saline [PBS]). For plating primary cells, cells were washed in cold complete medium (RPMI 1640 with L-glutamine and 10% FBS, 50 U/mL penicillin, 50 µg/mL streptomycin).

### Flow cytometry

One million bone marrow and spleen cells were resuspended in 50 µL antibody cocktail in cold FACS buffer and stained in the dark for 30 min on ice. Following three washes in FACS buffer, secondary streptavidin staining took place in PBS for 20 min in the dark on ice. Following three washes in FACS buffer, cells were either analyzed immediately using a BD FACSCanto, or fixed in 3% PFA and analyzed within 24 hours. Positive and negative gates were set using unstained or single-stained BD CompBeads (BD Biosciences, San Jose, CA).

### Immunization of LMP1/2A mice

Mice were immunized with 100 µg TNP_24_-KLH (Biosearch Technologies, Novato, CA) in CFA s.c. For germinal center analysis, spleen was isolated at Days 7 post-immunization. For serum ELISA, blood was drawn from anesthetized mice at Day 0, 7, 14 and 35 following immunization and sera separated by centrifugation. Mice were boosted after Day 50 with 50 ug of TNP_24_-KLH in sterile 1× PBS i.p.

### Histology

For hematoxylin and eosin (H&E) staining, spleen was isolated and processed as described [34]. For immunofluorescence, tissue was cryopreserved in Tissue-Tek OCT Compound (Redding, CA), snap frozen in a bath of ethanol and dry ice and stored at −80°C. Sections 5–6 µm thick were air dried for 10 min then fixed in ice cold acetone for 10 min. Sections were allowed to dry and rehydrated in a humid chamber for 20 min. Sections were blocked for 60 min with 10% goat serum, 5% bovine serum albumin (BSA) in 1× PBS, followed by washes with PBS. Incubations with primary antibody diluted in blocking buffer for 60 min were followed by washes in 1× PBS. Sections were incubated with secondary antibody diluted in blocking buffer for 30 min, washed twice and mounted with Fluoromount-G (Southern Biotech, Birmingham, AL).

### ELISA

To quantitate total serum immunoglobulin, plates were coated with isotype-specific purified antibodies. To quantitate TNP-specific IgG1, plates were coated with 50 µg/mL TNP_11_-BSA (Biosearch Technologies, Novato, CA). For IgG1 affinity, plates were coated with TNP_2_-BSA and TNP_11_-BSA. Following blocking with 3% BSA in PBS, serial dilutions of serum or an anti-TNP IgG1 standard (BD Biosciences) in blocking buffer in triplicate were incubated overnight at 4°C. Plates were washed multiple times with 1× TBST, and incubated with a biotinylated isotype-specific Ig, followed by washing and incubation with a streptavidin-conjugated HRP. Plates were developed with TMB (BioFX, Eden Prairie, MN) and stopped with StopSolution (BioFX, Eden Prairie, MN) and read at 450 nm on a Wallac Victor2 counter. IgG1 titers were calculated from the line generated from standards of a known calculation. Background subtracted (corrected) OD values were used to calculate the ratio of TNP_2_ (high affinity) to TNP_11_ (total) binding IgG1. For isotypes, corrected OD is shown as a percentage of the wildtype corrected OD.

### ELISPOT

For plasma cell ELIspots, immunized mice were boosted at Day 50 or later with 50 µg TNP-KLH i.p., and splenocytes and bone marrow isolated at day 7. Cells were treated with erythrocyte lysis buffer, washed and plated in B cell medium (RPMI 1640 with L-glutamine and 10% FBS, 50 U/mL penicillin, 50 µg/mL streptomycin, 50 µM β-mercaptoethanol) in serial dilutions (starting with 4×10^5^/well) on plates previously coated with 50 µg TNP_11_-BSA, and incubated for 18 hours at 37°C. IgG1-expressing ASC were revealed with streptavidin-AP and spots were counted using an ImmunoSpot (Cellular Technology LTD).

### Cell proliferation

Resting splenic CD43^−^ B cells were isolated from single cell suspensions by magnetic column (Miltenyi Biotec, Auburn, CA). Cells were incubated in B cell medium and stimulated with recombinant mouse IL-4 at 5 ng/mL (eBioscience, San Diego, CA), anti-CD40 at 10 µg/mL (eBioscience, San Diego, CA), goat anti-mouse anti-IgM F(ab′)_2_ at 10 µg/mL (Southern Biotech, Birmingham, AL) or LPS (Sigma-Aldrich). The concentrations of these reagents and the timepoint used had been previously optimized. Cells were incubated for 48 hours at 37°C, and 1 µCi ^3^[H]-thymidine was added for the last 18 hours of culture before the cells were harvested for analysis of thymidine uptake.

### RT-PCR

Total RNA was extracted from resting CD43^−^ splenic B cells using the RNeasy RNA Extraction kit (Qiagen) and cDNA was prepared with the High Capacity cDNA Reverse Transcription Kit (Applied Biosystems). Real-time PCR was performed and data analyzes as described previously [53]. Primer sequences for TRAF2, TRAF3 and HPRT are available at http://mouseprimerdepot.nci.nih.gov. The difference between the gene expression in transgenic compared to WT mice backgrounded to HPRT expression (ΔΔC_T_) was used to determine the relative gene expression in transgenic B cells compared with wildtype B cells, and fold change was calculated by 2^−ΔΔCT^ [66].

### Immunoblots

Purified B cells were lysed in modified RIPA buffer (0.1 M Tris-HCl pH 8.2, 0.15 M NaCl, 2% SDS, 1% NP40 alternate, 0.5% Na-deoxycholate, 0.01 M NaF, 0.002 M Na_3_VO_4_, 0.002 M phenylmethylsulfonyl floride, 0.01 M DTT) with protease and phosphatase inhibitor cocktails (Roche Diagnostics). Control lysates included CHO-K1 cells and CHO-K1/hTRAF2 (CHO-K1 cells transfected with hTRAF2 plasmid from Addgene, #20229). DNA and nucleic acid were digested with Benzonase nuclease (Sigma-Aldrich). Lysates were cleared and heated for 10 minutes at 72°C and then electrophoretically separated by 10% SDS-PAGE. Protein was transferred to Immobilon-P membrane (Millipore), blocked with 5% BSA in 1× TBST, and probed for TRAF2, TRAF3 and GAPDH. Membranes were incubated with IRDye secondary antibodies in blocking buffer for 1 h at room temperature, and imaged using a LiCor Odyssey Fc scanner and LiCor Image Studio Software (v2.0, Lincoln, NE). Boxes were manually placed around each band of interest, which returned near-infrared fluorescent values of raw intensity with intra-lane background subtracted [67]. TRAF2 and TRAF3 signal was normalized to GAPDH for each sample. The relative expression of TRAF2 and TRAF3 for each sample relative to wildtype was calculated by (normalized signal_sample_/normalized signal_wildtype_)*1.

## Supporting Information

Figure S1 **Lymphocyte frequencies are similar in LMP1/2A mice and controls.** Single cell suspensions from different lymphoid organs of 8 week old WT, LMP1, LMP2A or LMP1/2A mice were surface stained with indicated antibodies and analyzed by flow cytometry. Frequencies of the live indicated cell population are shown in (A–F). (A) Recirculating mature B cells (IgM^+^/IgD^+^) in bone marrow. (B) B1a cells (CD5^+^/CD43^+^), B1b cells (CD5^−^/CD43^+^) in spleen. (C) CD4^+^ or CD8^+^ T cells in spleen. (D) Total B cells (B220^+^/IgM^+^) in lymph nodes. (E) Follicular (FO) B cells (IgM^+^/IgD^+^) or marginal zone (MZ) B cells (IgM^+^/IgD^−^) in lymph nodes. (F) CD4^+^ or CD8^+^ T cells in spleen. *n*>4 mice per genotype for all experiments; *, *P*<0.05, Student's *t* test.
(TIF)

Figure S2 **TRAF2 levels in purified B cells using a different antibody.** (A) Whole cell lysates from purified splenic B cells of WT (n = 7), LMP1 (n = 8), LMP2A (n = 8) and LMP1/2A (n = 7) animals and CHO-K1/hTRAF2 cells were probed for TRAF2 using an antibody from Santa Cruz (C-20). A representative immunoblot is shown. (B) Quantification of TRAF2 signal normalized to GAPDH and wildtype TRAF2 signal is shown as an average of all mice from each genotype by LI-COR Odyssey analysis. LMP1 bars have been placed first as LMP1 B cells showed the largest TRAF2 increase. Data are represented as mean ± standard error. **, P<0.01 by one way ANOVA.
(TIF)
