# Supplementary figures and images for: Epstein-Barr Virus LMP2A Reduces Hyperactivation Induced by LMP1 to Restore Normal B Cell Phenotype in Transgenic Mice
Source: PLoS Pathog. 2012 Apr 19;8(4):e1002662. doi: 10.1371/journal.ppat.1002662 (PMC3334893; doi:10.1371/journal.ppat.1002662)

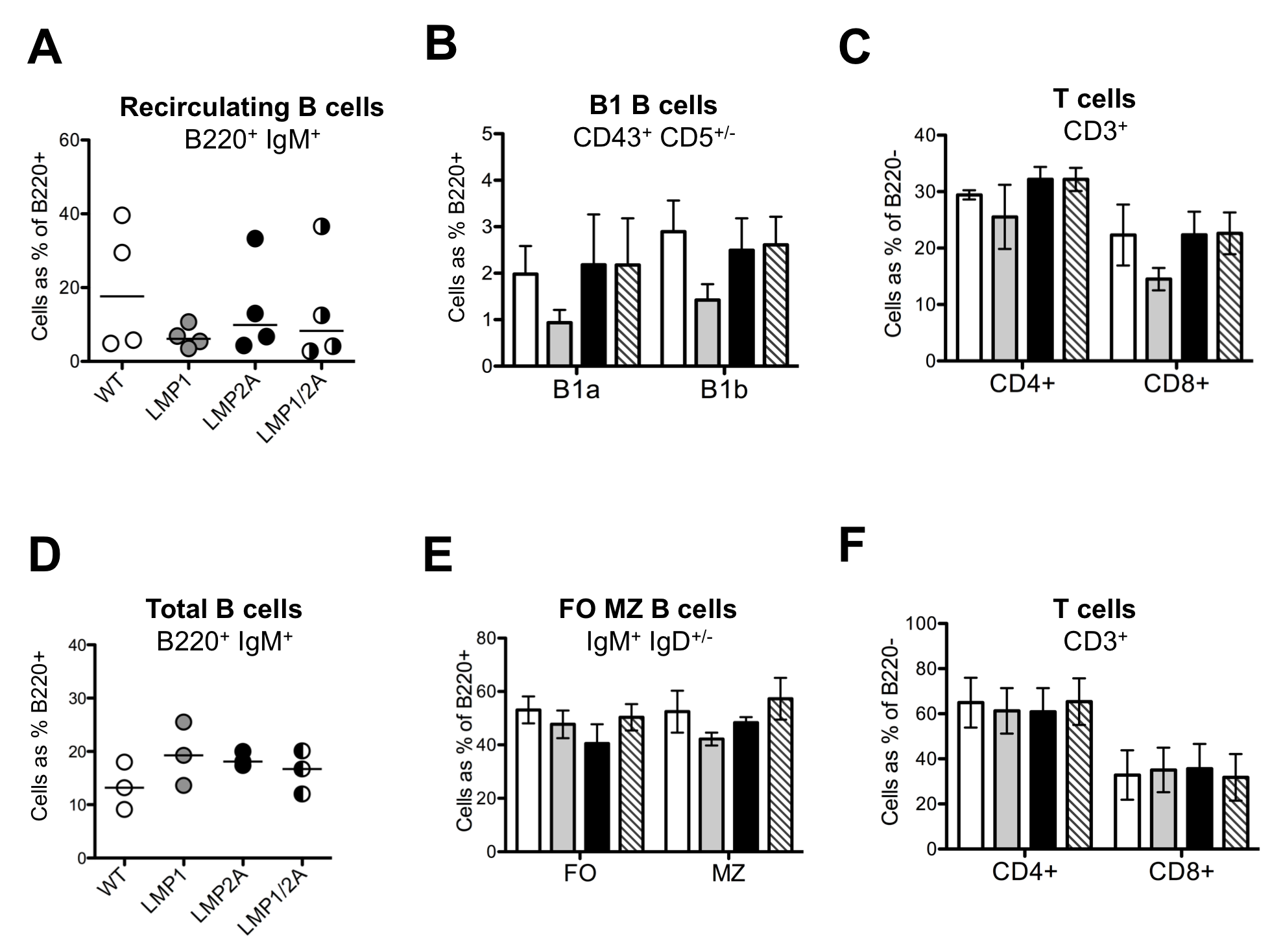

Supplement: Figure S1 — Lymphocyte frequencies are similar in LMP1/2A mice and controls. Single cell suspensions from different lymphoid organs of 8 week old WT, LMP1, LMP2A or LMP1/2A mice were surface stained with indicated antibodies and analyzed by flow cytometry. Frequencies of the live indicated cell population are shown in (A–F). (A) Recirculating mature B cells (IgM+/IgD+) in bone marrow. (B) B1a cells (CD5+/CD43+), B1b cells (CD5−/CD43+) in spleen. (C) CD4+ or CD8+ T cells in spleen. (D) Total B cells (B220+/IgM+) in lymph nodes. (E) Follicular (FO) B cells (IgM+/IgD+) or marginal zone (MZ) B cells (IgM+/IgD−) in lymph nodes. (F) CD4+ or CD8+ T cells in spleen. n>4 mice per genotype for all experiments; *, P<0.05, Student's t test. (TIF) [file ppat.1002662.s001.tif]

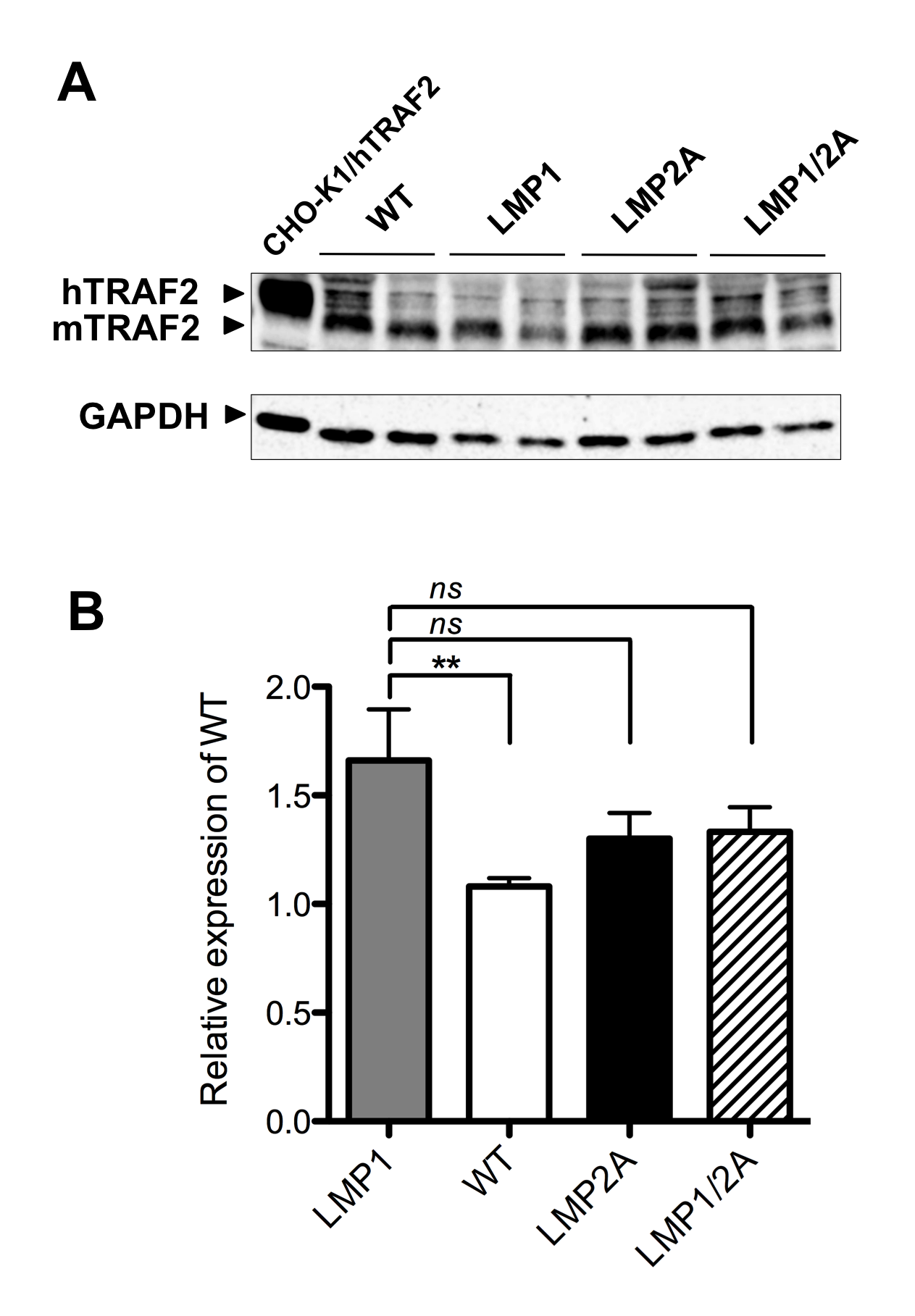

Supplement: Figure S2 — TRAF2 levels in purified B cells using a different antibody. (A) Whole cell lysates from purified splenic B cells of WT (n = 7), LMP1 (n = 8), LMP2A (n = 8) and LMP1/2A (n = 7) animals and CHO-K1/hTRAF2 cells were probed for TRAF2 using an antibody from Santa Cruz (C-20). A representative immunoblot is shown. (B) Quantification of TRAF2 signal normalized to GAPDH and wildtype TRAF2 signal is shown as an average of all mice from each genotype by LI-COR Odyssey analysis. LMP1 bars have been placed first as LMP1 B cells showed the largest TRAF2 increase. Data are represented as mean ± standard error. **, P<0.01 by one way ANOVA. (TIF) [file ppat.1002662.s002.tif]
